# Effects of Host and virus related factors on Interferon-α+ribavirin and Pegylated-interferon+ribavirin treatment outcomes in Chronic Hepatitis C patients

**DOI:** 10.1186/1743-422X-8-234

**Published:** 2011-05-17

**Authors:** Madiha Akram, Muhammad Idrees, Shamail Zafar, Abrar Hussain, Sadia Butt, Samia Afzal, Irshad-ur Rehman, Ali Liaqat, Sana Saleem, Muhammad Ali, Azeem Butt

**Affiliations:** 1 Molecular Virology Lab, National Centre of Excellence in Molecular Biology, 87-West Canal Bank Road Thokar Niaz Baig Lahore-53700, University of the Punjab, Lahore, Pakistan; 2 Department of Medicine, Lahore Medical and Dental College Lahore, Pakistan

## Abstract

**Background:**

Current standard therapy commonly followed for chronic Hepatitis C Virus (HCV) in Pakistan is interferon alpha plus ribavirin combination therapy (IFN α/ribavirin) and pegylated interferon plus ribavirin (PegIFN/ribavirin). PegIFN/ribavirin has increased rate of sustained virological response than standard IFN α/ribavirin therapy. Objective of current study was to analyze rate of early and delayed response to antiviral treatment as well as rate of relapse response in patients following standard treatment IFN α/ribavirin and in patients following pegylated interferon treatment.

**Methods:**

Baseline serum samples of 153 patients enrolled for IFN α/ribavirin and 50 patients for PegIFN/ribavirin were collected. After total RNA extraction, genotyping was and HCV RNA viral load was done. Subsequently HCV RNA viral load was estimated at 4 weeks of treatment, at 12 weeks, at 24 or 48 weeks and finally after 6 months follow up period. All the data was statistically analyzed using fisher's exact test.

**Results:**

Total 86 patients out of 153 patients following conventional IFN α/ribavirin therapy completed treatment and 69% of them showed Rapid Virological Response (RVR). Whereas 50 patients following PegIFN/ribavirin treatment completed treatment and 80% of them achieved RVR. Total 64 out of 86 patients following IFN α/ribavirin therapy completed follow up period and 53.5% of them achieved Sustainded Virologcal Response (SVR). Forty-five out of total 50 patients who received PegIFN/ribavirin treatment completed 6 months follow up period and among these 70% achieved SVR. SVR rates were significantly associated with RVR (p < 0.001), age (p < 0.001) and gender (p < 0.01)

**Conclusions:**

Rate of sustained virological response can be determined by factors like rapid virological response and age since they share significant association with one another. More over rate of SVR was more prominent in males than in females.

## Background

Hepatitis C virus, causative agent of chronic liver disease has affected approximately 175 million people worldwide which make up to almost 3% of the world's populations and 3 to 4 million new cases adds up to this figure annually [1]. Chronic HCV infection progresses towards more severe outcomes in the form of cirrhosis and hepatocellular carcinoma (HCC) [2]. Since protective vaccine against HCV is not available therefore successful treatment of HCV is much needed. The standard therapy for HCV treatment consist of subcutaneous intake of 3 MU/ml of IFN α three times per week [3] plus oral intake of 10 mg/day/kg of the body weight of ribavirin daily for 24 weeks [4]. This standard therapy is not ideal as success rate is not hundred percent in all patients. A modified version of treatment consists of pegylated interferon (80 MU/ml once a week) plus ribavirin, administered for 12 to 72 weeks [5].

Different people respond differently to this α-interferon treatment regimen and various terminologies are used for these responses to therapy. When HCV RNA becomes undetectable (< 50 IU/ml) after treatment completion, the response is characterized as end of treatment response (ETR), treatment response is said to be sustained virological response (SVR) if HCV RNA remain undetectable at the end of follow up (6 months post treatment completion), but if it reappears after 6 months of follow up period than it is termed as relapsed. However patients with a detectable HCV RNA throughout the treatment are non responders (NR) [6]. Another recently studied emerging predictor of treatment response, rapid virological response (RVR), defined as a negative HCV RNA after four weeks of antiviral treatment [7,8]. A 2 log decrease in HCV RNA after 12 weeks of treatment is defined as early virological response (EVR). Breakthrough response (BT) is the type of response when HCV RNA remains undetectable during the treatment but reappears before the end of treatment [9-11].

Various viral and host related factors are responsible for such diversity in responses. HCV genotype and the pre-treatment viral load influence the antiviral response rates and this rate is higher for genotype 2 and 3 than for genotype 1 [12]. Genotype 3a that is reported as the most respondent to combination therapy is the most frequent HCV genotype circulating in Pakistan [13]. Another reported factor that determines the rate of SVR is the presence or absence of RVR [7]. Among host related factors, age and sex are very important as younger age and female sex decrease the risk of disease progression [14]. Furthermore treatment response is affected by age, as younger patients (age < 40 years) are associated with significantly higher rate of SVR than older aged patients [7].

There are studies that have analyzed the rate of response to IFN-α plus ribavirin treatment in Pakistan along with various factors that might predict rate of SVR [7], however these studies do not describe the effect of different treatment strategies such as standard IFN-α plus ribavirin combination therapy and PegIFN plus ribavirin therapy on treatment response. Therefore the aim of this study was to study the effect of different factors such as sex, age, pre-treatment viral load and genotype in two groups of patients treated either with IFN-α plus ribavirin or PegIFN plus ribavirin. This study would be useful in tailoring the treatment according to the patient's demographic status and in establishing which treatment should be followed for better results.

## Methods

### Patients Enrolled

Patient's demographic data and samples were collected from 203 patients with chronic HCV infection from July 2009 to July 2010 at Ghurki Trust Hospital Lahore. Out of these 203 patients, 152 patients (first group) received standard HCV treatment of Interferon (3 Million units per ml) 3 injections per week and Ribavirin 10 mg/day/kg of the body weight, While 50 patients (second group) received pegylated interferon (PegIFN 80 million units per ml) 1 injection per week and ribavirin (10 mg/day/kg of the body weight). Patients infected by HCV genotypes None-1 and HCV-1 (or with co-infection with HCV-1) received therapy for 24 weeks and 48 weeks respectively. Total 67 patients were excluded from final data analysis of the study since they were either unwilling to participate in the study at some stage, sara samples were insufficient or lacked the requested information regarding demographic characteristics. All the 136 eligible patients were anti-HCV ELISA and serum HCV RNA positive.

### Quantitative detection of HCV RNA

Serum samples were collected from enrolled patients before treatment, during treatment (at 4 weeks and 12 weeks) and at the end of treatment. All the serum samples were analyzed for quantitative detection of HCV RNA titer using SmartCycler II real time (Cephid, USA).

### HCV Genotyping

HCV genotyping was done on pre-treatment sera samples using the genotyping method described previously [15].

### Patient's monitoring

Patient's viral load was evaluated before treatment at 1 month, 3 months, 6 months (non-1 genotypes), 12 months (HCV-1) and at month 6 post treatment. Undetectable HCV RNA titer (< 250 IU/ml) after first month and third month of treatment was described as RVR and EVR response respectively. Undetectable HCV RNA at month 3 of treatment but reappearance of HCV RNA at end of treatment was defined as breakthrough response (BTR). HCV RNA less than 250 IU/mL at 6 months of treatment was defined as end of treatment response (ETR). Undetectable HCV RNA (< 250 IU/ml) 6 months after treatment cessation was defined as sustained virological response (SVR). Patients with detectable HCV RNA throughout the treatment were classified as non responders while ETR patients with detectable HCV RNA after 12 months were defined as relapsers.

### Statistical evaluation

SPSS version 16.0 was used to analyze all variables (age, sex, genotypes, pre-treatment viral load, type of treatment, rate of response). Means with standard deviation and percentage frequencies of variables were calculated where needed. Significant association amongst variables was analyzed by calculating p-value using Fisher's Exact t-test. P-value less than 0.05 were indicative of significant association.

## Results

### Demographic and clinical characteristics

**Figure 1:**
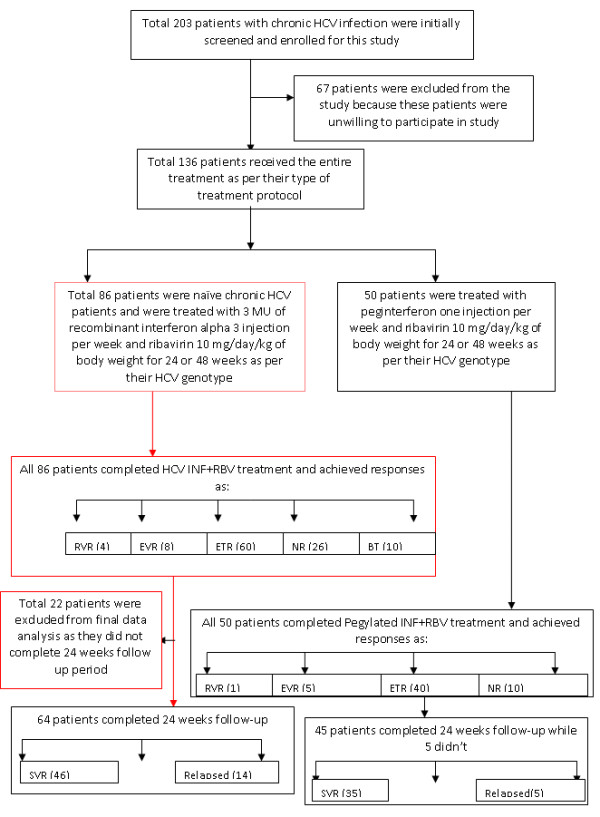
**Patients disposition and treatment responses**.

Figure 1 shows the patients disposition and treatment response. Out of total 203 enrolled subjects, 104 were males and 98 were females. All of the patients received treatment except 67 patients who either could not completed treatment for 6 months or they were unwilling to participate in the study therefore were excluded from final data analysis. Total 136 patients received treatment for complete treatment duration of (6 months or 12 months). Demographic and virological characteristics of these studied patients are summarized in table 1. For data analysis, these patients were divided into two groups based on the type of treatment they received.

**Table 1:** Demographic and virological characteristics of enrolled patients who completed the entire treatment course (N = 136).

Characteristics	Number (%)
**Sex**	
Male N (%)	69 (50.7)
Female N (%)	67 (49.3)

**Pre-treatment HCV RNA level**	
>200,000 IU/ml N (%)	112 (82.4)
<200,000 IU/ml N (%)	24 (17.6)

**Genotypes N (%)**	
3a	111 (81.6)
3b	1 (0.7)
1a	6 (4.4)
3a/1a	4 (2.9)
3a/1b	2 (1.5)
2b	1 (0.7)
Un typable	11 (8.1)

**Characteristics**	**Age Range-years ± SD**

**Mean Age (Y)**	17-60 ± 10.02

N: number; IU: International units; ml: millilitre; SD, standard deviation;

Table 2 indicates distribution of genotypes in enrolled patients. Genotype 3a was the predominant (more than 81%) genotype in patients subjected to these two different types of treatments. Genotype 3a was the predominant genotype in both the groups of patients. Total 86 out of these 136 patients received 3 MU/ml of IFN-α administered thrice a week along with 10 mg/day/kg of the body weight of ribavirin administered orally. Remaining 50 patients were subjected to 80 MU/ml of Pegylated IFN administered once a week along with same ribavirin dose for recommended duration based on its genotype. In first group patients, 64 patients completed 6 months post treatment follow up period whereas 22 patients in this group did not cooperate in the study by providing requested information after treatment completion. Of the second group, 45 out of 50 patients completed the follow up period and only 5 patients lost interest in study.

**Table 2:** Distribution of HCV genotypes in patients subjected to different types of treatments (N = 136)

*HCV Genotypes*	*Treatment type*	
	
	IFNα & RBV (%)	PegIFN & RBV (%)
3a	78(90.6)	33 (66)
3b	None	1 (2)
1a	1 (1.2)	5 (10)
3a/1a	None	4 (8)
3a/1b	None	2 (4)
2b	1 (.7)	none
Untypable	6 (6.9)	5 (10)
**Total**	**86**	**50**

HCV: hepatitis C virus; INF: interferon; Peg: pegylated; RBV: ribavirin

### HCV Treatment response analysis

Table 3 translates the effects of different variables such as sex, age, genotypes, treatment type and pre-treatment viral load on treatment responses. SVR rates were found higher significantly higher (p < 0.01) in male patients compared to female patients. No significant difference was seen in RVR, EVR and BTR in both sexes. Treatment response was different in different age groups. A significantly higher (p < 0.01) SVR was seen for patients less than 20 years (66.7%) and 21-30 years (76.2%) patients groups. A low SVR rate (44.4%) was observed for age group 50-60 years and above group. Similar results were seen for other type of responses such as RVR, EVR and BTR in different age groups. Rate of treatment response was also analyzed HCV genotype-wise. No significance difference was observed for RVR, EVR, BTR and ETR. Even SVR rates were same in infected patients by Non-1 (59.7%) or 1 & mixed genotypes (58.3%). Rates of ETR and SVR in patients treated with PegIFN and ribavirin was significantly higher than in patients received IFN-α and ribavirin combination therapy. Similarly relapse rates were found significantly higher in patients treated with IFN-α and ribavirin therapy than in patients treated with Pegylated IFN/ribavirin therapy. Effect of pre-treatment viral load on the rate of response rates was also studied. Patients having pre-treatment viral load < 200,000 IU/ml showed a slightly lower rate of ETR and SVR than patients having viral titer > 200,000 IU/ml, however, the difference was not significant (p 0.14).

**Table 3:** Rates of responses in different sexes, ages, genotypes, treatment types and viral titres (N = 136)

Factor	Total Treated	RVR (%)	EVR (%)	BTR (%)	ETR (%)	NR (%)	SVR (%)
**SEX**							
**Male** **Female**	69 67	3(4.3) 2(2.9)	7(10.1) 6(8.9)	4(5.8) 6(8.9)	52(75.4) 48(71.6)	17(24.6) 19(28.3)	48(69.6) 33(49.3)

**AGE-years**							
**<20**	6	0	0	0	5(83.3)	1(16.7)	4(66.7)
**21-30**	42	1(2.4)	4(9.5)	2(4.8)	35(83.3)	7(16.7)	32(76.2)
**31-40**	43	2(4.7)	6(13.9)	5(11.6)	28(65.1)	15(34.9)	21(48.8)
**41-50**	36	2(5.6)	2(5.6)	2(5.6)	27(75)	9(25)	19(52.8)
**51-60 & above**	9	0	1(11.1)	1(11.1)	5(55.6)	4(44.4)	4(44.4)

**GENOTYPE**							
**None-1**	124	5(4)	12(9.7)	9(7.3)	92(74.2)	32(25.8)	74(59.7)
**1 & Mixed**	12	0	1(8.3)	1(8.3)	8(66.7)	4(33.3)	7(58.3)

**TREATMENT**							
**INF+RBV**	86	4(4.7)	8(9.3)	10(11.6)	60(69.8)	26(30.2)	46(53.5)
**PEGINF+RBV**	50	1(2)	5(10)	0	40(80)	10(20)	35(70)

**PRE-TREATMEN**							
**VIRAL LOAD**							
**<200 IU/mL**	112	4(3.6)	13(11.6)	9(8)	81(72.3)	31(27.7)	65(58)
**>200 IU/mL**	24	1(4.2)	0	1(4.2)	19(79.1)	5(20)	16(66.7)

**TOTAL**	136	5(3.6)	13(9.6)	10(7.4)	100(73.5)	36(26.5)	81(59.6)

RVR: Rapid virological response; EVR: Early Virological response; BTR: Breakthrough response; ETR: End of treatment response; NR: Non-responders; SVR: Sustained virological response; INF: Interferon; PEG-INF: Pegylated interferon; RBV: ribavirin; IU/International units

### Factors associated with SVR rates

Table 4 shows several factors that are associated significantly with SVR rates that can be utilize before treatment for the prediction of treatment outcome. Among these RVR and age are the strongest predictors of treatment destiny.

**Table 4:** Association of gender, pre treatment viral load, RVR and age with rate of SVR in patients following HCV antiviral treatment

*Variable*	*Treated *	*SVR (N)*	*SVR rate *	*P value <*
	*patients *		*(%)*	
	*(N)*			
**Gender**				
Male	69	48	69.6	0.01
Female	67	33	49.2	
**Pre treatment viral load**				
≤ 200,000 IU/ml				
> 200,000 IU/ml	24	16	66.7	0.14 (NS)
	112	65	58.0	
**RVR**				
Present	5	5	100	0.001
Absent	131	76	58	
**Age**				
≤ 30 years	48	36	75	0.001
> 30 years	88	44	50	

RVR: rapid virological response; SVR: Sustain virological response; NS: Non-significance; IU: International units

## Discussion

Interferon (IFN) plus ribavirin is a routine treatment for chronic HCV infection in Pakistan. It comprises 3 MIU given thrice a week along with ribavirin 800 to1200 mg administered per day. Previous data shows that rate of SVR raises to 38-43% with standard IFN α plus ribavirin therapy [16]. A modified version of treatment is PegIFN α plus ribavirin, it is the current treatment depending upon factors like HCV genotype, baseline viral load, and initial virological response to therapy [17], however this type of treatment is not common in Pakistan due to high cost of treatment. It is administered only once a week and have 10 times longer half life of plasma than the conventional IFN [18].

In the current study we have checked several host and viral factors on treatment outcome in patients who received both types of therapies. According to the results of our study, rate of SVR was significantly higher (p < 0.01) in patients received pegylated interferon compared to non-pegylated interferon. Despite of this high rate of SVR, most of our patients received standard IFN-α plus ribavirin therapy due to cost factor as the pegylated interferon is at least 25 times expensive than non-pegylated interferon therefore is beyond the reach of poor people in underdeveloped country like Pakistan [19]. It has already been reported that rate of SVR with PegIFN-α and ribavirin is comparatively high with genotype 2 and 3 (80%) than genotype 1 or 4 (40-50%) [5]. Our study support the findings of Shin and colleagues [20] that SVR rate is high in genotype 3 (a&b) compared to HCV genotype 1a when the current standard therapy is used for treatment of HCV. Shin and colleagues [20] further reported in their study that genotype 1 is a risk factor for relapse response. Our study confirmed this observation of Shin and colleagues [20] as relapse response was about two times higher in genotype 1 infected patients (40%) compared to relapse rates seen in patients harbouring genotype 3 HCV infections. In addition to cost, several side effects have also been reported to be associated with PegIFN plus ribavirin therapy such as depression and anemia [5] therefore conventional IFN plus ribavirin remains the gold standard for treatment of chronic HCV [21] in underdeveloped countries like Pakistan.

Age is another factor that might determine the treatment response. It has been reported recently that patient age greater than 50 years increases the risk of relapse [19]. Our study supports this observation of others as in our study, rate of relapsers increased as the age progresses. Relapse rate was lowest (8.6%) in patients with an age ranging from 20 to 30 years and rose to 33.3% in patients with an age above 50 years. SVR rate was 91.4% in patients with age less than 30 and decreased to 66.7% with an age above 50 years. Our study further supports the idea that age must be considered as an important associated factor while recommending treatment to chronic HCV patients since SVR rate significantly differed between different age groups (p-value < .05).

Recently Shin and co-workers (19) has reported a higher base line titer (≥ 200,000 IU/ml) as an independent risk factor for higher relapse rate. In yet another study low pretreatment viral load was pointed out as predictor of the achievement of improved SVR rate as SVR was significantly higher in patients that had baseline viral load less than 200,000 IU/ml [7]. However, we were unable to observe such an association between pre-treatment viral load and treatment response. Relapse rate decreased to only 8.6% in our patients with baseline viral titer lower than 200,000 IU/ml. However SVR rate in patients with baseline viral titer in both cases (> or < 200,000 IU/ml did not significantly differed (p-value > .05).

Another important prognostic factor of the attainment of SVR is achievement of RVR. A significantly association was seen between RVR and SVR in the current study (p < .001) as our all patients who had RVR had also achieved SVR. Unfortunately the observed RVR rate is very low as only our 3.6% patients had this type of rapid response at week 4 of treatment. The RVR response has already been established as an important predictor of SVR in more than 85% cases [7].

HCV infection progresses slowly in females than in males [14]. Unlike to several other studies (7, 16), the observed SVR rate was significantly higher in males than in females (p < 0.01).

## Conclusions

Our study with reference to previous studies confirms younger age and rapid virological response as important factors for the achievement of sustained virological response. Our study further established that male patients have a greater chance of achieving SVR responses.

## Competing interests

The authors declare that they have no competing interests.

## Authors' contributions

MA and MI conceived the study, participated in its design and coordination and gave a critical view of manuscript writing. MA performed and analyzed the results. AH and SB participated in results analysis. SZ provided clinical data of patients and monitored the patients throught the study. BK, SA, IR, LA, AB, SS and MA participated in data analysis. All the authors read and approved the final manuscript.
